# Effect of high-dose rosuvastatin loading before percutaneous coronary intervention in Chinese patients with acute coronary syndrome: A systematic review and meta-analysis

**DOI:** 10.1371/journal.pone.0171682

**Published:** 2017-02-23

**Authors:** Ziliang Ye, Haili Lu, Qiang Su, Wenqin Guo, Weiran Dai, Hongqing Li, Huafeng Yang, Lang Li

**Affiliations:** 1 Department of Cardiology, the First Affiliated Hospital of Guangxi Medical University, Nanning, Guangxi, China; 2 Department of Orthodontics, the Affiliated Dental Hospital of Guangxi Medical University, Nanning, Guangxi, China; Worcester Polytechnic Institute, UNITED STATES

## Abstract

**Background:**

Acute coronary syndrome (ACS) is an important disease threatening human life and health. Many studies have shown that the loading dose of atorvastatin can significantly improve the prognosis of patients with ACS, and reduce the mortality. However, this conclusion is not consistent. Thus, we aimed to evaluate the effect of high-dose rosuvastatin loading before percutaneous coronary intervention (PCI) in Chinese patients with ACS using a meta-analysis based on a systematic review of published articles.

**Methods:**

We systematically reviewed published studies, evaluating the effect of high-dose rosuvastatin loading before percutaneous coronary intervention in Chinese patients with ACS. The retrieval time is limited from inception to 2 November 2016, and the retrieved databases included PubMed, Embase, the Cochrane Library, Web of Science, CBM, CNKI, the VIP database and the Wang Fang database. Two researchers independently assessed the quality of the included studies and then extracted the data. Stata 11.0 was used for data analysis.

**Results:**

In total, 11 articles, which included 802 patients, were included in our meta-analysis. Among these patients, 398 patients were in the high-dose group (20 mg/day) and 404 patients were in the conventional dose group (10 mg/day). Meta-analysis results showed that compared with the conventional dose group: 1) The loading dose of rosuvastatin can significantly reduce the hs-CRP level after PCI, including at 24 hours (SMD = -0.65, 95%CI -0.84 ~ -0.47, P = 0.000), 48 hours (SMD = -0.40, 95%CI -0.68 ~ -0.11, P = 0.006), and four weeks (SMD = -1.64, 95%CI -2.01 ~ -1.26, P = 0.000). 2) The loading dose of rosuvastatin can significantly reduce the levels of LDL-C and cTnT, including the level of LDL-C at 30 d after PCI (SMD = -0.89, 95%CI -1.10 ~ -0.69, P = 0.000), and the level of cTnT at 24 h after PCI (SMD = -1.93, 95%CI -2.28 ~ -1.59, P = 0.000), and increase the level of HDL-C at 48 h after PCI (SMD = 0.61, 95%CI 0.34 ~ 0.88, P = 0.000). 3) The loading dose of rosuvastatin can significantly reduce the levels of TG and TC, including the level of TG at 30 d after PCI (SMD = -0.94, 95%CI -1.17 ~ -0.71, P = 0.000), the level of TC at 48 h after PCI (SMD = -0.35, 95%CI -0.68 ~ -0.01, P = 0.043), and the level of TC at 30 d after PCI (SMD = -0.77, 95%CI -0.98 ~ -0.56, P = 0.000).

**Conclusions:**

Our systematic review and meta-analysis showed that, compared with the conventional dose, the loading dose of rosuvastatin was more beneficial to patients with ACS in China and is suitable for clinical application. Due to the limitations of the quality and quantity of included articles, this conclusion still needs to be confirmed by multicenter clinical trials.

## Introduction

Acute coronary syndrome (ACS) [1, 2, 3, 4] is a syndrome caused by decreased blood flow in the coronary arteries, such that part of the heart muscle is unable to function properly or dies. The mainly symptom of ACS is chest pain, which often radiates to the left arm or angle of the jaw, is pressure-like in character and is often associated with nausea and sweating. The results of many studies have indicated that ACS is typically caused by three problems: ST elevation myocardial infarction (STEMI, 30%), non-ST elevation myocardial infarction (NSTEMI, 25%), or unstable angina (38%) [5]. In recent years, with the improvement of people's living standards and the change in diet and living environment, the incidence of coronary heart disease has increased, which can seriously harm human health [6, 7]. Depending on the incomplete statistics, the cost of hospitalization for coronary artery atherosclerosis in the United States has reached 10 billion 400 million USD, and approximately 1 million 200 thousand ACS patients are hospitalized annually [8]. In China, the incidence of ACS and mortality rate have also shown an increasing trend with every year [9].

Percutaneous coronary intervention(PCI) [10, 11] is one of the important methods for the treatment of coronary heart disease. In particular, the extensive use of drug eluting stents significantly reduces the incidence of restenosis after PCI and cardiovascular events. In the United States, approximately 1 million 500 thousand people receive PCI treatment annually, and approximately 5–30% of patients exhibit perioperative myocardial infarction (PMI). It was found that the incidence of PMI was approximately the same as spontaneous myocardial infarction [12]. An increasing number of experiments have confirmed that during PCI operation, in addition to mechanical damage, some substances that act on blood vessels and have biological activities are also released. They enter the microcirculation via the blood vessels, resulting in vascular contraction and inflammation, endothelial dysfunction, myocardial ischemia and necrosis. All of these factors can cause the heart muscle to be damaged within a short time period, which can lead to complications such as myocardial injury and infarction in the perioperative period.

Statin [13] drugs not only function in lowering blood fat, but they also have multiple effects independent of lipid regulation, including anti-inflammation, anti-atherosclerotic, anti-platelet aggregation, inhibition of thrombosis, maintenance of plaque stability, improvement in endothelial function, etc. In addition, statins can reduce the expression of adhesion molecules, prevent the aggregation of inflammatory molecules and improve the function of small blood vessels [14]. At the present, many studies [15, 16] have found that early use of statins in patients with ACS can reduce the incidence of major adverse cardiovascular events (MACE) caused by interventional therapy and improve the prognosis of patients.

Many results [17, 18, 19] have shown that compared with atorvastatin, the effect of rosuvastatin on lowering blood fat was stronger. Early application of rosuvastatin can effectively regulate blood lipids, reverse atherosclerotic plaquea and reduce the atherosclerosis inflammatory index, which reduce cardiovascular events after PCI. In addition, the incidence of adverse events was less than that of similar drugs. As a new potent statin, both animal experiments and clinical trials have shown that rosuvastatin demonstrates high clinical efficacy. Up to now, the 40 mg dosage form of rosuvastatin has not yet entered China, and the maximum dose of rosuvastatin is 20 mg per day in China [20], which differs from American and European countries (the maximum dose of rosuvastatin is 40 mg or 80 mg per day). At the present, many studies in China have indicated that the loading dose of atorvastatin can improve the prognosis of patients with ACS. However, this conclusion is inconsistent. In addition, those trials are small sample trials, and the number of patients included was small, resulting in poor clinical reliability. Thus, we performed a meta-analysis to evaluate the effect of high-dose rosuvastatin loading before PCI in Chinese patients with ACS and to provide a reference for clinical applications.

## Materials and methods

### Literature search

According to the statement of the preferred reporting items for Systematic Reviews and Meta-Analyses, two researchers independently searched the published articles that investigated the effect of high-dose rosuvastatin loading before PCI in Chinese patients with ACS. The retrieved database included PubMed, Embase, the Cochrane Library, Web of Science, CBM, CNKI, the VIP database and the Wang Fang database. The retrieval time was limited from inception to November 2, 2016, and the research object was limited to Chinese people. Relevant keywords related to Rosuvastatin in combination as MeSH terms and text words(“Rosuvastatin Calcium” or “Calcium, Rosuvastatin” or “Crestor” or “Rosuvastatin” or “ZD4522” or “ZD 4522”) were used in combination with words related to Acute Coronary Syndrome(“Acute Coronary Syndromes” or “Coronary Syndrome, Acute” or “Coronary Syndromes, Acute” or “Syndrome, Acute Coronary” or “Syndromes, Acute Coronary”). The retrieval language was limited to Chinese and English. Furthermore, reference articles of the extracted articles were also retrieved. When multiple reports of the same study were present, we used the most recent publication and supplemented it. All analyses were based on previously published studies, and thus no ethical approval or patient consent was required.

### Study selection

We identified studies that prospectively evaluated the effect of high-dose rosuvastatin loading before PCI in Chinese patients with ACS. Inclusion criteria: ①All patients met the diagnostic criteria for acute coronary syndrome; ②The study was limited to randomized controlled trials (randomized controlled trials, RCTs); ③The subjects for our study were Chinese populations; ④The loading dose of rosuvastatin was 20 mg per day, and the conventional dose of rosuvastatin was 10 mg per day; ⑤The usage time of rosuvastatin was not limited; ⑥The article should provide sufficient data for analysis.

Exclusion criteria: ①Retrospective, non-randomized trials; ②Semi-randomized controlled trials, in which the grouping method of the participants in the experiment was not strictly random; ③Articles with incomplete or erroneous data; ④Patients with allergies to rosuvastatin; ⑤Patients with serious infections, endocarditis, pulmonary infarction, blood system diseases, peripheral vascular occlusive disease or malignant tumor; ⑥In the past 6 months, there was surgery or trauma, and a history of cerebral hemorrhage; ⑦Patients with aortic dissection.

### Data extraction

The contents of the retrieved studies were reviewed by two researchers according to prior search methods. To determine whether the article mets the inclusion criteria, a standard data extraction table was used for data extraction. Data to be extracted included basic data of the subjects (First author, Average age, Year of publication), dose of rosuvastatin used (loading dose group and conventional dose group) and observation index. If there was a lack of necessary data or some content to be clarified in the articles, an effort was made to try to make contact with the study authors, and if the necessary data to analyze was still unavailable, this article was excluded.

### Statistical analyses

We used Stata software, Version 11.0 (Stata Corp, College Station, Tex) to pool and analyze the results from the individual studies. Pooled results were recorded as the standardized mean difference (SMD), and presented with 95% CIs with two-sided P-values. P < 0.05 indicated that the difference was statistically significant. Heterogeneity of the inclusion study was assessed using the I^2^ test, which assessed the appropriateness of pooling the individual study results. When I^2^ < 50%, the heterogeneity of the study was considered small; when I^2^>50%, the heterogeneity of the study was considered substantial. Funnel plots were ued to investigate the sources of heterogeneity. Furthermore, meta-regression analysis was performed to explore heterogeneity [21].

## Results

**Fig 1:**
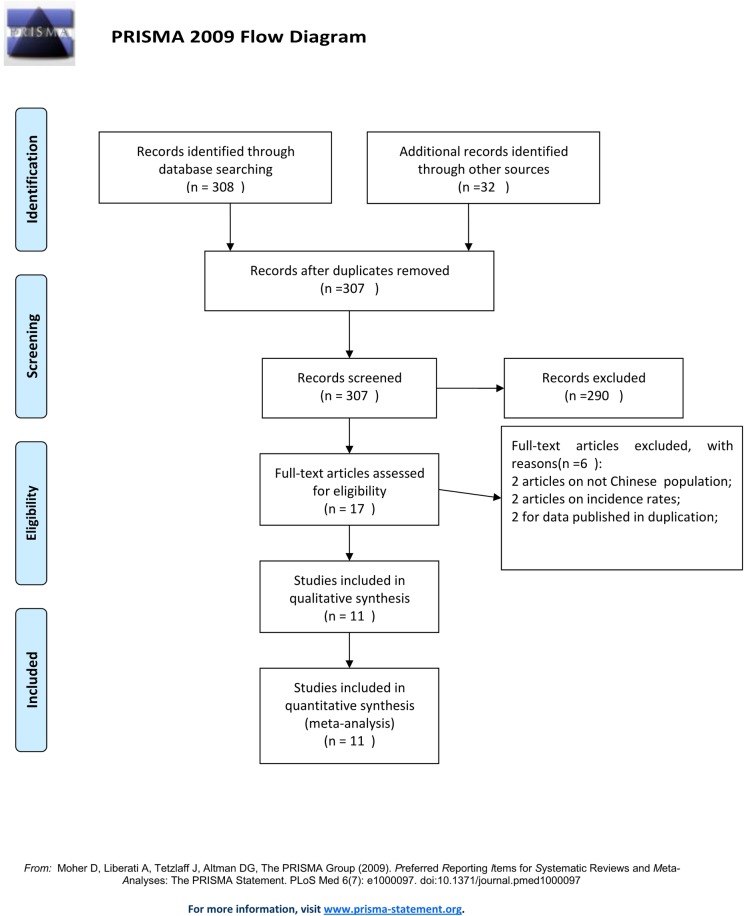
Flowchart of the selection strategy and inclusion/exclusion criteria in the current meta-analysis. Flowchart of the selection strategy and inclusion/exclusion criteria in the current meta-analysis.

Our study searched the relevant articles in Chinese and English published from inception to November 2, 2016 in a Chinese population. In total, data from 11 articles [22, 23, 24, 25, 26, 27, 28, 29, 30, 31, 32], which consisted of 802 patients, were included into our meta-analysis. Among them, 398 patients were placed in the high-dose group (20 mg/day) and 404 patients were placed in the conventional dose group (10 mg/day)(Fig 1). The basic information of each included study is shown in **Table 1**.

**Table 1:** Characteristics of studies included in meta-analysis.

Author	year	country	Sample number		Average age (years)		Use method of loading dose group	Use method of conventional dose group	outcome Measures
			R	C	R	C			
Wu HB[22]	2013	china	50	50	59.28±9.02	56.28±9.81	Oral(20 mg/day)	Oral(10 mg/day)	hs-CRP, TG, TC, LDL-C
Xu CZ[23]	2013	china	45	45	53~84	53~84	Oral(20 mg/day)	Oral(10 mg/day)	hs-CRP, TG, TC, LDL-C, HDL-C
Zou Y[24]	2012	china	43	37	56±10.1	56±10.6	Oral(20 mg/day)	Oral(10 mg/day)	hs-CRP, TG, TC, LDL-C, HDL-C
Chen QY[25]	2016	china	30	31	20~75	20~75	Oral(20 mg/day)	Oral(10 mg/day)	hs-CRP, TG, TC, LDL-C
Jiao YG[26]	2014	china	33	39	59.3±11.8	60.9±10.7	Oral(20 mg/day)	Oral(10 mg/day)	hs-CRP, TC, LDL-C,TnT
Li H[27]	2014	china	30	30	65.2±2.4	66.3±2.6	Oral(20 mg/day)	Oral(10 mg/day)	hs-CRP
Gui W[28]	2014	china	26	26	54.2±7.8	53.2±6.8	Oral(20 mg/day)	Oral(10 mg/day)	hs-CRP, TG, TC, LDL-C, HDL-C
Yang Y[29]	2015	china	20	20	59±8.65	59±8.65	Oral(20 mg/day)	Oral(10 mg/day)	TnT
Shi YB[30]	2013	china	20	20	69.10±11.95	70.90 ±13.72	Oral(20 mg/day)	Oral(10 mg/day)	TG, TC, LDL-C
Liao BH[31]	2013	china	70	70	58.91±12.52	61.38±14.33	Oral(20 mg/day)	Oral(10 mg/day)	hs-CRP
Luo J[32]	2012	china	31	36	58.3±11.8	60.6±8.8	Oral(20 mg/day)	Oral(10 mg/day)	TnT

Loading dose of Rosuvastatin group = R; Conventional dose Rosuvastatin group = C; hypersensitive C reactive protein = hs-CRP; triglyceride = TG; total cholesterol = TC; Low-density lipoprotein = LDL-C; High Density Lipoprotein = HDL-C; Cardiac troponin T = TnT

### Literature quality evaluation

Of the eleven articles, one article [29] used the method of a random number table, seven articles [22, 23, 27, 28, 30, 31, 32] referred to a random method but did not give a specific description, and the randomization strategy was not clear in three articles [24, 25, 26]. The hidden distribution of the eleven articles [22, 23, 24, 25, 26, 27, 28, 29, 30, 31, 32] was low; nine articles [22, 23, 24, 26, 27, 28, 29, 30, 32] used a random single-blind method, but the blind method of two articles [25, 31] was unclear. The incomplete outcome data of the eleven articles [22, 23, 24, 25, 26, 27, 28, 29, 30, 31, 32] was low. The inclusion criteria and exclusion criteria of one article [23] was unclear, and the other biases of the 11 articles [22, 23, 24, 25, 26, 27, 28, 29, 30, 31, 32] was low. The literature quality score is shown in **Table 2**.

**Table 2:** Assessment of Methodological Quality of Included Studies.

Study	random allocation	Hidden distribution	Blind method	Incomplete Outcome Data	Selective reporting of results	Other bias	quality grade
Wu HB[22]	mentioned random	Low	Single-blind	Low	Low	Low	A
Xu CZ[23]	mentioned random	Low	Single-blind	Low	No clear	Low	B
Zou Y[24]	No clear	Low	Single-blind	Low	Low	Low	B
Chen QY[25]	No clear	Low	No clear	Low	Low	Low	B
Jiao YG[26]	No clear	Low	Single-blind	Low	No clear	Low	B
Li H[27]	mentioned random	Low	Single-blind	Low	Low	Low	A
Gui W[28]	mentioned random	Low	Single-blind	Low	Low	Low	A
Yang Y[29]	random number table	Low	Single-blind	Low	Low	Low	A
Shi YB[30]	mentioned random	Low	Single-blind	Low	Low	Low	A
Liao BH[31]	mentioned random	Low	No clear	Low	Low	Low	B
Luo J[32]	mentioned random	Low	Single-blind	Low	Low	Low	A

### Change of hs-CRP after PCI

**Fig 2:**
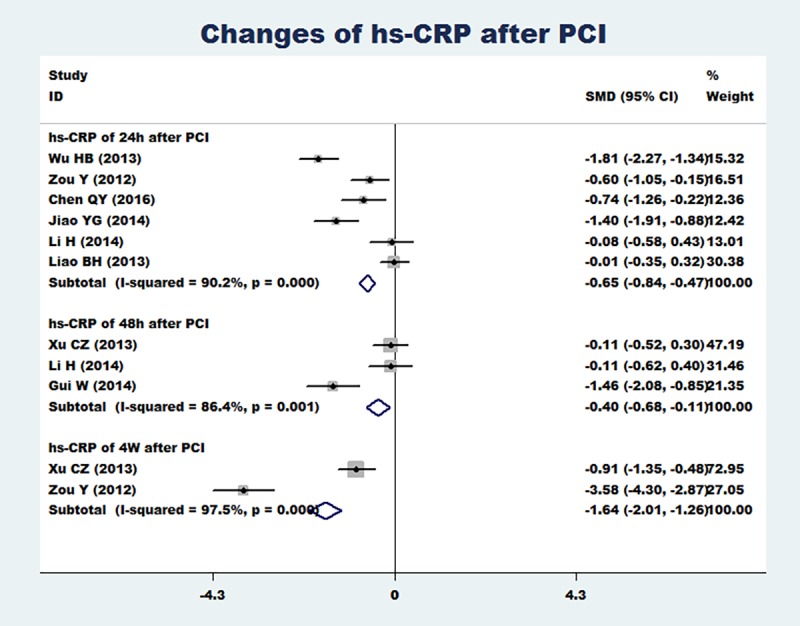
Change of hs-CRP after PCI(including the change of hs-CRP at 24 h after PCI, the change of hs-CRP at 48 h after PCI and the change of hs-CRP at 4 weeks after PCI). Change of hs-CRP after PCI(including the change of hs-CRP at 24 h after PCI, the change of hs-CRP at 48 h after PCI and the change of hs-CRP at 4 weeks after PCI).

**Fig 3:**
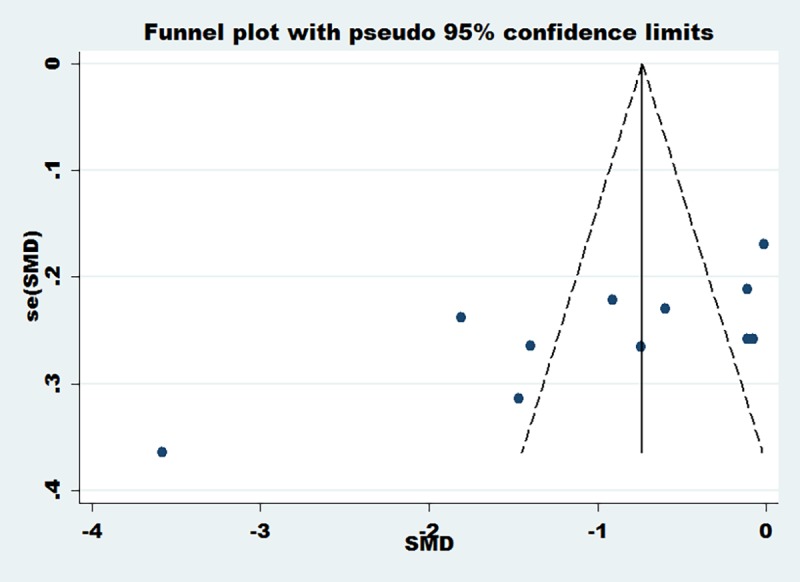
Funnel plots of the change of hs-CRP after PCI. Funnel plots of the change of hs-CRP after PCI.

Six articles [22, 24, 25, 26, 27, 31] reported the change of hs-CRP at 24 h after PCI, three articles [23, 27, 28] reported the change of hs-CRP at 48 h after PCI and two articles [23, 24] reported the change of hs-CRP at four weeks after PCI. Meta-analysis results showed that compared with the that of the conventional dose group, the loading dose of rosuvastatin can significantly reduce the hs-CRP level after PCI, including at 24 hours (SMD = -0.65, 95%CI -0.84 ~ -0.47, P = 0.000), 48 hours (SMD = -0.40, 95%CI -0.68 ~ -0.11,P = 0.006) and 4 weeks (SMD = -1.64, 95%CI -2.01 ~ -1.26, P = 0.000)(Fig 2). Because there was obvious heterogeneity for overall analysis (I^2^>50%), publication bias was assessed using funnel plots. On the basis of the funnel plots ((Fig 3), there may be a specific publication bias in the included articles.

### Change of LDL-C,HDL-C and cTnT after PCI

**Fig 4:**
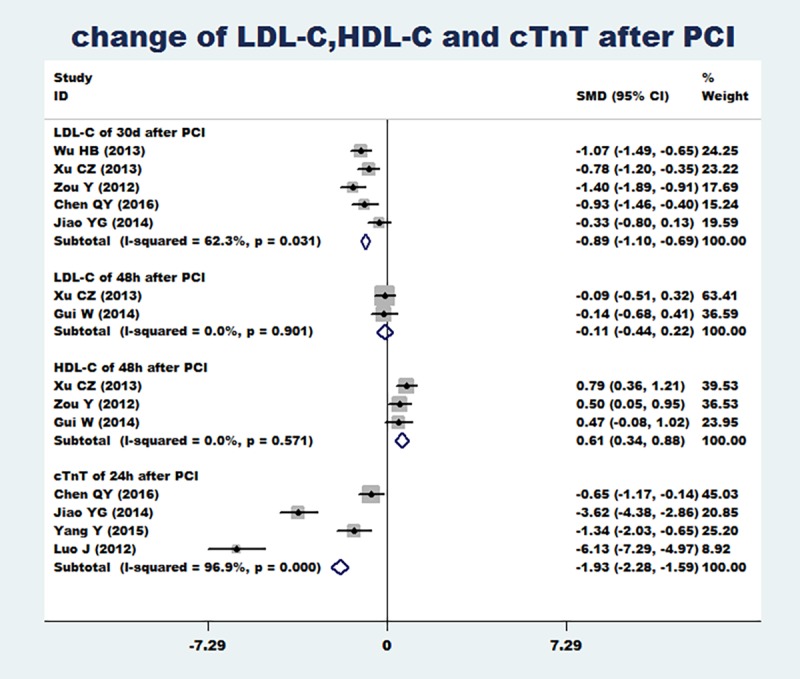
Changes of LDL-C,HDL-C and cTnT after PCI (including the change of LDL-C at 48 h after PCI, the change of LDL-C at 30 d after PCI, the change of HDL-C at 48 h after PCI and the change of cTnT at 24 h after PCI). Changes of LDL-C,HDL-C and cTnT after PCI (including the change of LDL-C at 48 h after PCI, the change of LDL-C at 30 d after PCI, the change of HDL-C at 48 h after PCI and the change of cTnT at 24 h after PCI).

**Fig 5:**
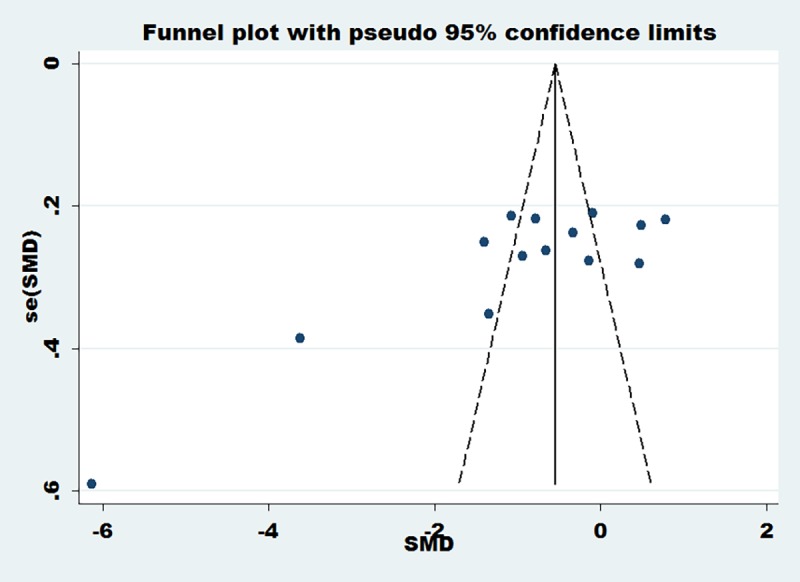
Funnel plots of the change of LDL-C,HDL-C and cTnT after PCI. Funnel plots of the change of LDL-C,HDL-C and cTnT after PCI.

Two articles [23, 28] reported the change of LDL-C at 48 h after PCI, five articles [22, 23, 24, 25, 26] reported the change of LDL-C at 30 d after PCI, three articles [23, 24, 28] reported the change of HDL-C at 48 h after PCI and four articles [25, 26, 29, 32] reported the change of cTnT at 24 h after PCI. Meta-analysis results showed that compared with the conventional dose group, the loading dose of rosuvastatin can significantly reduce the level of LDL-C and cTnT after PCI, including the level of LDL-C at 30 d after PCI (SMD = -0.89, 95%CI -1.10 ~ -0.69, P = 0.000), the level of cTnT at 24 h after PCI (SMD = -1.93, 95%CI -2.28 ~ -1.59, P = 0.000), but cannot reduce the level of LDL-C at 48 h after PCI (P = 0.513)(Fig 4). In addition, the loading dose of rosuvastatin can significantly increase the level of HDL-C at 48 h after PCI (SMD = 0.61, 95%CI 0.34 ~ 0.88, P = 0.000). The heterogeneity of the included articles was obvious (I^2^>50%), and funnel plots were used to assess publication bias. Funnel plots ((Fig 5)) showed that there might be some heterogeneity in the included articles.

### Change of TG and TC after PCI

**Fig 6:**
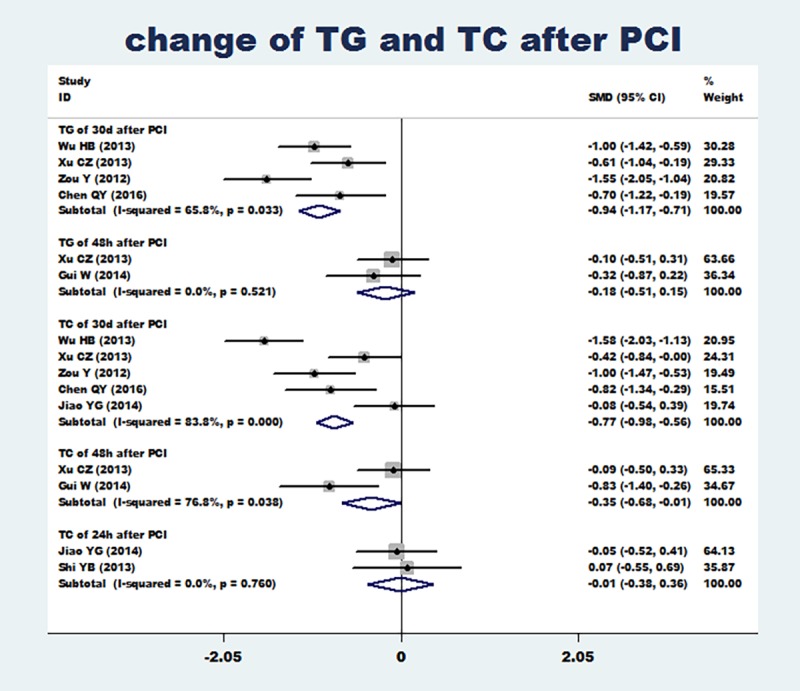
Changes of TG and TC after PCI (including the change of TG at 48 h after PCI, the change of TG at 30 d after PCI, the change of TC at 24 h after PCI, the change of TC at 48 h after PCI and the change of TC at 30 d after PCI). Changes of TG and TC after PCI (including the change of TG at 48 h after PCI, the change of TG at 30 d after PCI, the change of TC at 24 h after PCI, the change of TC at 48 h after PCI and the change of TC at 30 d after PCI).

**Fig 7:**
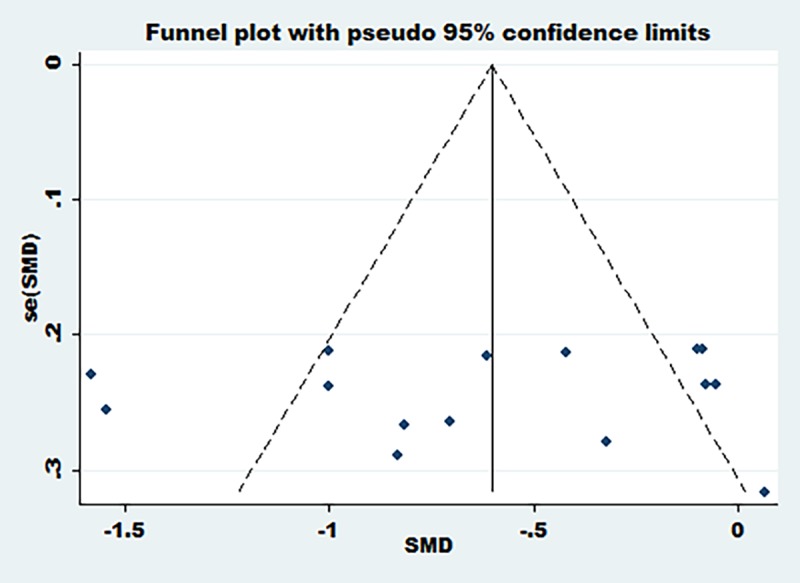
Funnel plots of the change of TG and TC after PCI. Funnel plots of the change of TG and TC after PCI.

Two articles [23, 28] reported the change of TG at 48 h after PCI, four articles [22, 23, 24, 25] reported the change of TG at 30 d after PCI, two articles [26, 30] reported the change of TC at 24 h after PCI, two articles [23, 28] reported the change of TC at 48 h after PCI and five articles [22, 23, 24, 25, 26] reported the change of TC at 30 d after PCI. Meta-analysis results showed that compared with the conventional dose group, the loading dose of rosuvastatin can significantly reduce the level of TG and TC, including the level of TG at 30 d after PCI (SMD = -0.94, 95%CI -1.17 ~ -0.71, P = 0.000), the level of TC at 48 h after PCI (SMD = -0.35, 95%CI -0.68 ~ -0.01, P = 0.043) and the level of TC at 30 d after PCI (SMD = -0.77, 95%CI -0.98 ~ -0.56, P = 0.000), but cannot reduce the level of TG at 48 h after PCI and the level of TC at 24 h after PCI(Fig 6). The heterogeneity of the included articles was obvious (I^2^>50%), and funnel plots were used to assess publication bias. Funnel plots (Fig 7) showed that there might be some heterogeneity in the included articles.

### Publication bias

**Fig 8:**
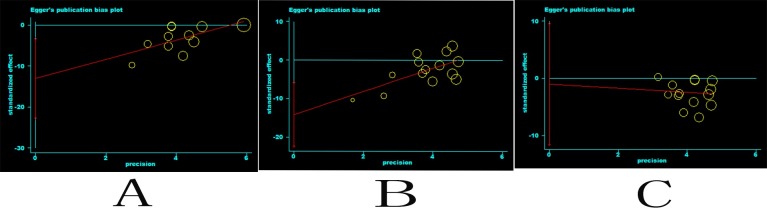
Funnel plots of hs-CRP, LDL-C,HDL-C, cTnT, TG and TC. Funnel plots of hs-CRP, LDL-C,HDL-C, cTnT, TG and TC.

Because the object of our study is a Chinese population, our meta-analysis did not have a subgroup analysis based on the population. From the funnel plots and I^2^ above, we found that there may be some bias in the included articles, and thus meta-regression analysis was performed to evaluate publication bias quantitatively. Egger’s test did not exhibit obvious publication bias under the change of TG and TC after PCI (P = 0.841) (**Fig 8C**), but Egger’s test demonstrated obvious publication bias under the change of hs-CRP after PCI (P = 0.014) (**Fig 8A**) and the change of LDL-C,HDL-C and cTnT after PCI (P = 0.003) (**Fig 8B**).

## Discussion

Acute coronary syndrome (ACS) [33, 34, 35] is a type of acute vascular event of atherosclerotic disease, that will often endanger the patient's life, and it has become one of the major diseases in developed countries and China. Despite modern medical standards and improvement in technology, there is still a high incidence of adverse cardiovascular events in patients with ACS. Previous research data indicate that the hospitalized mortality rate of ST segment elevation myocardial infarction is approximately 7%, while the hospitalized mortality rate of non-ST segment elevation acute coronary is approximately 3–5% [36, 37]. ACS has become one of the major diseases threatening human life and health, which have widely aroused the attention of clinicians.

The pathogenesis of ACS is very complex [38, 39], and its pathological mechanism is not yet completely clear. It may involve lipid metabolism, inflammation and immune response, endothelial cells, vascular smooth muscle cells and other cells. A remarkable curative effect has been achieved since PCI has been used in the clinic. In addition, PCI has become one of the most important treatment modalities for ACS patients [40]. In recent years, there have been a series of studies about the application of statins in the perioperative period of PCI worldwide, and these results suggested that the use of high-dose statins can improve the clinical prognosis of patients and reduce the incidence of adverse cardiovascular events after PCI [41, 42].

In our current meta-analysis, we found that compared with the conventional dose of rosuvastatin, the loading dose of rosuvastatin could significantly reduce the level of hs-CRP after PCI, including at 24 hours (SMD = -0.65, 95%CI -0.84 ~ -0.47, P = 0.000), 48 hours (SMD = -0.40, 95%CI -0.68 ~ -0.11, P = 0.006) and four weeks (SMD = -1.64, 95%CI -2.01 ~ -1.26, P = 0.000), suggesting that the loading dose of rosuvastatin could decrease the inflammatory index and reduce the incidence of postoperative infection, which is beneficial to the prognosis of patients after PCI. In addition, the levels of LDL-C and cTnT of the high-dose group were significantly lower than the conventional dose group. This difference was statistically significant (P<0.05), which indicated that the effect of the loading dose of atorvastatin on myocardial protective effect was stronger compared to the conventional dose group. Moreover, the loading dose of rosuvastatin can significantly reduce the levels of TG and TC, including the level of TG at 30 d after PCI (P = 0.000), the level of TC at 48 h after PCI (P = 0.043) and the level of TC at 30 d after PCI (P = 0.000). In summary, we speculated that the loading dose of rosuvastatin not only had a good short-term advantage, but that the long-term clinical therapeutic effect was also significant.

Many studies [43, 44] also indicated that in the treatment of patients with ACS, the loading dose of rosuvastatin was better than the conventional dose. Cay [45] performed a trial, and 299 patients were randomized to a rosuvastatin-treatment (n = 153) group and 146 patients were placed in to a no-treatment group. A forty mg loading dose of rosuvastatin was administered 24 h before PCI, and the CK-MB and cTnI levels were measured before and at 12 h after PCI. The incidence of CK-MB and cTnI elevation in the rosuvastatin group was significantly lower compared with the control group (0.7% vs. 11.0%, p<0.001 and 10.5% vs. 39.0%, p<0.001, respectively), and Cay indicated that high loading dose of rosuvastatin (40 mg/day) could effectively reduce the incidence of peri-procedural myocardial necrosis and infarction. In 2011, a 12-month follow-up trial was also performed by Yun KH [46]. In this study, 445 patients with ACS who underwent PCI were randomly assigned to receive no statin treatment before PCI (control group, n = 220) or to receive 40mg rosuvastatin loading before PCI (rosuvastatin group, n = 225), and cardiac death, non-fatal MI, non-fatal stroke and any ischemia-driven revascularization were assessed after 12 months. During the follow-up, major adverse cardiac events occurred in 20.5% of patients in the control group and 9.8% of patients in the rosuvastatin group (p = 0.002), and the incidence of death and non-fatal MI was significantly higher in the control group compared with loading dose of atorvastatin (p = 0.021). Finally, Yun demonstrated that high-dose rosuvastatin loading before PCI can significantly improve 12-month clinical outcomes in patients with ACS.

There are several limitations in our meta-analysis. First, positive results are most likely to be published, and publication bias cannot be completely excluded, and some publication bias was observed during this study. Second, a total of 11 randomized controlled studies were included in our meta-analysis, but most of the studies have some limitations, including the allocation method and implementation methods. In addition, the inclusion criteria and exclusion criteria of some articles were not obvious. Third, the object of our study was Chinese people, and did not include people from other countries and races. This conclusion is potentially only suitable for a Chinese population, and unsuitable for people from other regions. Therefore, if this conclusion applies to other people, then it should be interpreted with caution. Fourth, in the Chinese market, the highest dose of rosuvastatin is 20 mg per day. However, in Europe and the United States, the highest dose of rosuvastatin is 40 mg or 80 mg per day, and thus different doses may result in different effects. Finally, studies included in our meta-analysis were all published in Chinese Journals, so the quality of the articles may not be as high as studies published in English Journals, and some necessary information was unclear.

In conclusion, our meta-analyses suggested that compared with the conventional dose group, the loading dose of rosuvastatin was more beneficial to patients with ACS in China, making it suitable for clinical application. However, due to the limitations of the quality and quantity of the included articles, further large scale or even countrywide studies are needed.

## Supporting information

S1 File The original data. (XLS)

S2 File All of the articles of this meta-analysis. (RAR)

S3 File PRISMA 2009 checklist. (DOC)
